# Internal Hernia Through a Congenital Defect in Broad Ligament: A Rare and Elusive Cause of Intestinal Obstruction

**DOI:** 10.7759/cureus.24769

**Published:** 2022-05-06

**Authors:** Partha Nandi, Sudhir Jain, Akhil Kainth, Shivani Atri

**Affiliations:** 1 General Surgery, Employees' State Insurance Corporation (ESIC) Medical College and Hospital, Faridabad, IND

**Keywords:** case report, surgery, internal hernia, congenital broad ligament defect, small intestinal obstruction

## Abstract

Internal hernias through a defect in the broad ligament is an uncommon cause of intestinal obstruction and most often the finding is intra-operative. What makes it rare is when the hernia occurs through a congenital defect in the broad ligament. We present the case of a 48-year-old female patient operated on an emergency basis for intestinal obstruction and intra-operatively the cause was identified to be a herniated ileal loop through a defect in the broad ligament. There was no history of any prior abdominal surgery or any interventions and all of her children were born by normal vaginal delivery, making it a case of congenital broad ligament defect. The postoperative period was uneventful and she was discharged in good health.

## Introduction

Protrusion of abdominal viscera through an opening within the boundaries of the peritoneum could be termed Internal Hernia [1], accounting for 0.6% to 5.8% of the cases of intestinal obstruction [2]. Though it can be both through congenital or acquired defects, the rarest ones are those herniating through congenital defects and presenting later in life. Congenital factors include spontaneous rupture of cystic structures developing from the remnants of the mesonephric Müllerian ducts and developmental peritoneal defect around the uterus [3]. Such cases often present with features of acute obstruction and if left untreated may proceed towards strangulation and may even cause gangrene of the herniating viscera. We present a case of one of the rarest forms of congenital internal hernia through a broad ligament defect in a 48-year-old female discovered during exploratory laparotomy for intestinal obstruction along with a review of the literature.

## Case presentation

A 48-year-old female presented in emergency with chief complaints of colicky pain abdomen associated with on and off nonprojectile vomiting for 15 days along with absolute constipation for the last two days. There was no history of any comorbid illnesses or prior abdominal surgeries. All of her children were born by normal vaginal delivery.

On examination, she was having tachycardia and showing signs of moderate dehydration and rest was normal. The abdomen was soft but distended with no guarding or rigidity. There were visible and palpable bowel loops in the lower abdomen. On auscultation, hyperperistaltic bowel sounds were heard. An initial x-ray was done showing features of small bowel obstruction. All baseline blood investigations were normal except for hyponatremia. CECT's whole abdomen was planned. But, she continued to deteriorate and was taken up for emergency laparotomy. 

Initially, she was resuscitated with IV fluids, IV antibiotics, continuous nasogastric tube aspiration, and other supportive measures. Once hyponatremia was corrected, she underwent exploratory laparotomy.

**Figure 1:**
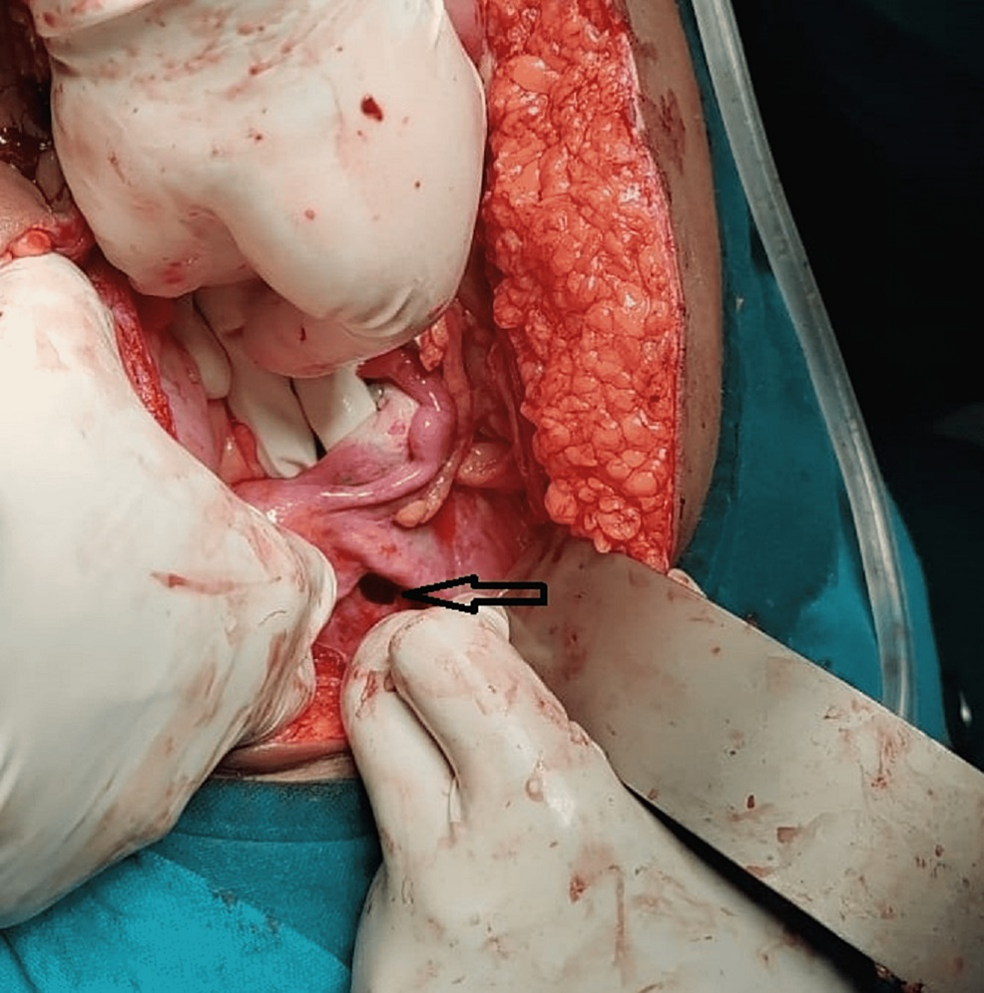
Defect in left broad ligament as indicated by the arrow.

**Figure 2:**
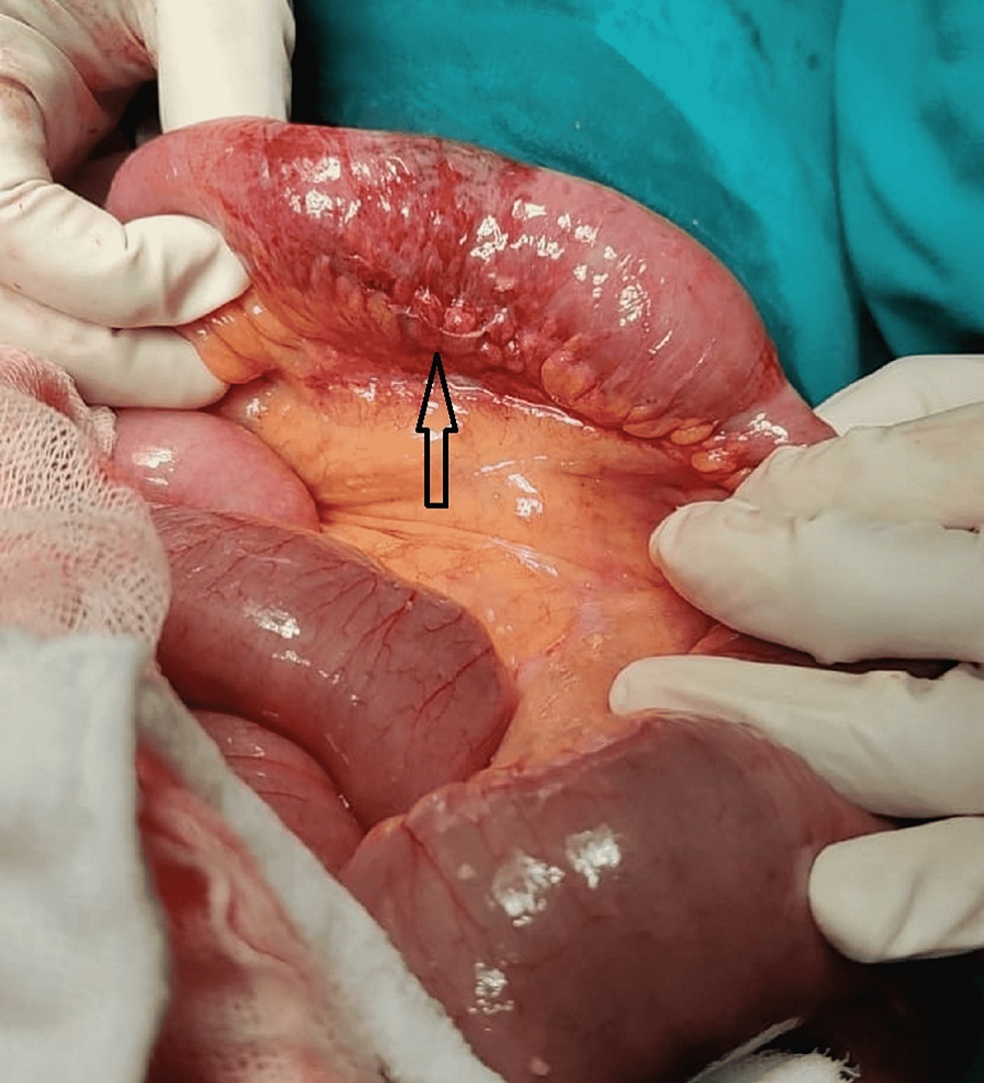
Non-viable segment of ileum (marked by the arrow).

During laparotomy, the proximal small gut was grossly distended. On, further exploration, distal ileal loops were herniating through a defect in the left Broad ligament (Figure 1). Opinion was taken from the Gynaecology team to confirm the location of the defect. The other side was explored to rule out any such defect. As approximately 10 cm of the herniated loop was found to be nonviable (Figure 2), resection and primary anastomosis were done and the Broad ligament defect was closed with interrupted sutures. Postoperatively patient recovered well.

Postoperative period was uneventful and she recovered well.

## Discussion

Embryology

The joining of the Mullerian ducts is followed by the formation of broad ligaments during the embryological developmental phase and this fusion leads to the formation of the female pelvic organs. A broad ligament is, actually, a fusion of the two layers of peritoneum [4].

Anatomy

Different parts of the broad ligament are named based on the structures contained between the two layers of peritoneum forming the broad ligament. The largest portion is the mesometrium extending laterally from the entire surface of the uterus. The mesosalpinx drapes over both the uterine tubes and the mesovarium attach the anterior portion of each ovary to the posterior part of the broad ligament [4].

Congenital internal abdominal hernias can either be retroperitoneal or formed through congenital anomalous openings or defects. Retroperitoneal hernias include paraduodenal (30%-53%), Winslow's foramen (8%), paracecal (6%), and intersigmoid hernias (5%), while those occurring through congenital openings can be, further, classified as transmesenteric (5%-10%), broad ligament (4%-7%) or transomental hernias (1%-4%) [5]. While most recorded in children, congenital internal hernias are very rare in adults.

Though congenital internal hernias are rare, this pathology needs to be considered while working up a patient with acute symptoms of bowel obstruction, with no external hernias [5]. While investigations like an abdominal x-ray and USG whole abdomen might provide some clue towards the diagnosis of intestinal obstruction other than history and a clinical examination, a contrast CT scan might delineate the pathology. But the most important diagnostic modality is Exploration under Anesthesia.

The first reported case of such type of hernia, similar to our report, was by Quain in 1861 [6]. Cilley in 1986 divided broad ligament defects into three types: type 1 defects occurring caudal to the round ligament , type 2 defects those above the round ligament of the uterus are type 2 defects, and type 3 defects are those occurring in the mesoligamentum teres of the uterus [7]. Lelardi et al. [8] cited a case of herniation of small bowel through a congenital defect in the broad ligament. Alberto et al. [9] cited two similar cases of small bowel herniations through congenital broad ligament defect in one of their publications.

Being a phenomenon of rare occurrence, citations and articles regarding small bowel herniation through congenital broad ligament defect are rare. Management of such condition is always surgical which might include an exploratory laparotomy or a surgeon might proceed with a laparoscopic intervention. The basis of surgery is reduction of content and primary closure of the defect. If needed resection of the strangulated or gangrenous bowel might be performed and in cases of peritonitis, the need for creating a diversion might arise. In some cases, the adnexa need to be excised out.

## Conclusions

Internal herniation of the small bowel through congenital broad ligament defect is the rarest finding and a diagnostic dilemma. A keen and quick clinical assessment aided by emergency radiological facilities helps in establishing an early and timed diagnosis. Such diagnosis needs quick surgical intervention and a delay might cause any permanent damage to the herniating structure. Hence, the management is always an emergency surgical exploration.
